# Correlation between the cephalometric measurements and acoustic properties of /s/ sound in Turkish

**DOI:** 10.1590/1678-7757-2019-0399

**Published:** 2020-04-27

**Authors:** Guzin Bilgin BUYUKNACAR, Aysegül GULEC

**Affiliations:** 1 Private Practice Department of Orthodontics Gaziantep Turkey Private Practice , Department of Orthodontics , Gaziantep , Turkey .; 2 Gaziantep University Department of Orthodontics Gaziantep Turkey Gaziantep University , Department of Orthodontics , Gaziantep , Turkey .

**Keywords:** Malocclusion, Speech disorders, Speech acoustics

## Abstract

**Objectives:**

To evaluate the acoustic properties of the /s/ sound in individuals with different occlusion types and to investigate relationships between these properties and cephalometric measurements.

**Methodology:**

Sixty patients were divided into three groups based on malocclusion. Group 1 included 20 patients (mean age: 14.85±2.01 years) with Class I skeletal and dental relationships. Group 2 included 20 patients (mean age: 13.49±1.78 years) with Class II skeletal and dental relationships. Group 3 included 20 patients (mean age: 12.46±2.62 years) with Class III skeletal and dental relationships. Cephalometric tracings were obtained from cephalometric radiographs. All included patients were native speakers of Turkish. The /s/ sound was selected for center of gravity analysis. Correlations between cephalometric values and acoustic parameters were also investigated.

**Results:**

The center of gravity of the /s/ sound had the lowest value in Group 2 (p<0.05). For the /s/ sound in Group 3, moderate positive correlations were found between center of gravity and Sella-Nasion to Gonion-Gnathion angle (p<0.05, r=0.444) Lower incisor to Nasion-B point (p<0.023, r=0.505), and Lower incisor to Nasion-B point angle (p<0.034; r=0.476). No correlation was found in other cephalometric measurements.

**Conclusions:**

The /s/ sound was affected by malocclusion due to the changing place of articulation. Therefore, referral to an orthodontist for malocclusion treatment especially patients with class III in the early period is suggested for producing acoustically ideal sound.

## Introduction

The reduction of speech problems after the completion of orthodontic treatment for speech impairments is important for both orthodontists and patients. Speech is presumed to be positively affected by malocclusion correction. Determination of the source of pretreatment speech problems in each patient is important to predict how speech might be affected after orthodontic treatment.

Speech is the most used form of communication and consists of sounds produced by interactions between the articulator and phonetic systems. Speech sounds are formed by partial or complete closure of the airway. Speech production involves four processes: respiration, phonation, resonance, and articulation. In the articulation phase, speech sounds are produced by dynamic movements of the tongue, lips, and teeth. Articulation disorders comprise 50–60% of speech disorders. ^1^ The degrees of Class II malocclusion, Class III malocclusion, overjet, open bite, and deep bite can influence speech; ^2 - 8^ individuals with one or more of these malocclusion types may produce normal speech by developing compensatory mechanisms. ^1^ The lingual alveolar sibilant /s/ is reportedly the sound most affected by dental and skeletal problems. ^9^

Studies investigating the effects of malocclusion on speech have included the assessment of voice recordings by speech-language pathologists, as well as direct conversation with such pathologists. Computer-aided sound analysis programs are increasingly preferred for sound analysis studies because they are objective, convenient, and repeatable. There are many studies investigating the relationship between speech disorders and malocclusions in the literature. However, the correlation between the spectral center of gravity values of /s/ sound and individual cephalometric measurement values have not been explicitly addressed. This study was performed to compare the effects of different skeletal malocclusion types on speech sounds using current examination methods. Furthermore, correlations between cephalometric values and center of gravity analysis were investigated to assess possible relationships between dentofacial anomalies and the /s/ sound.

## Methodology

### Study design and patient characteristics

The study design was prospective and involved 60 patients, who applied to the Faculty of Dentistry’s Department of Orthodontics at Gaziantep University for orthodontic treatment. The diagnosis of the patients was made by the same investigator (G.B.B) after clinical and cephalometric examination.

The inclusion criteria were as follows:

1. Class I dental and skeletal relationships;
2. Class II skeletal and dental relationships characterized by mandibular retrognathia and positive overjet;
3. Class III skeletal and dental relationships characterized by maxillary retrognathia and negative overjet.

The exclusion criteria were as follows:

1. Any neurological disorders, phonological problems, or articulation problems;
2. Any congenital anomalies (e.g., cleft lip and palate, anomalies related to the stomatognathic system).

Three groups were formed according to malocclusion types of the patients: group 1 consisted of 20 patients (8 men, 12 women; mean age: 14.85±2.01 years) with Class I dental and skeletal relationships, whose treatment was just finished at the faculty; group 2 consisted of 20 patients (9 men, 11 women; mean age: 13.49±1.78 years) with Class II skeletal and dental relationships who were at the beginning of their treatment; and group 3 consist of, 20 patients (8 men, 12 women; mean age: 12.46±2.62 years) with Class III skeletal and dental relationships again who were at the beginning of their treatment. All included patients were native speakers of Turkish. Approval for this study was obtained from the Gaziantep University Clinical Trials Ethics Committee (no. 2016/322). Written-informed consent was obtained from all patients and their guardians.

### Cephalometric and acoustic analysis

The anterior dental arch plays an essential role in the formation of sounds. For this reason, the sibilant fricative /s/ was assessed in this study. The Turkish words “saf,” “yas,” “sim,” and “mis” were placed in a carrier sentence (e.g., “Mehmet … dedi”).

Sound recordings were obtained before the initiation of orthodontic treatment for patients in all groups. Sound recording was performed in a dedicated soundproof room with acoustic insulation and a noise level of <30 dB in the Orthodontic Department of the Gaziantep University Faculty of Dentistry. For recording, a desktop computer (Asus Intel Core i5 4200U, Beitou District, Taipei, Taiwan) with an external sound card (Roland Ua-55 Quad Capture, Hamamatsu, Shizuoka, Japan) was used in conjunction with a condenser microphone (RODE NT1-A, Silverwater, NSW, Australia) positioned 10 cm away from the patient. The Audacity software (version 2.0.5, Boston, MA, USA) was used for audio recording; sounds were recorded at a sampling rate of 44,100 Hz, a 16-bit quantization level, in mono, and in “.wav” format.

**Figure 1:**
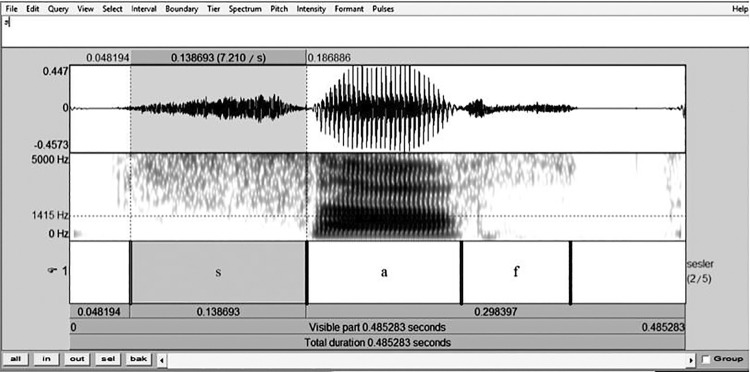
Onset and offset of fricative segmentation

Acoustic analysis was performed using the PRAAT (version 5.3.57, Phonetic Sciences, University of Amsterdam, Netherlands). Onset and offset of fricative segmentation were defined by visual inspection of the waveform and spectrogram ( Figure 1 ). Fricative onset was characterized by the point at which high-frequency energy first appeared, or by a rapid increase in zero crossings. Fricative offset was defined before the onset of vowel periodicity or as the absence of high-frequency energy. After segmentation, the spectral center of gravity (cog) was examined at the midpoint of the segment.

**Figure 2:**
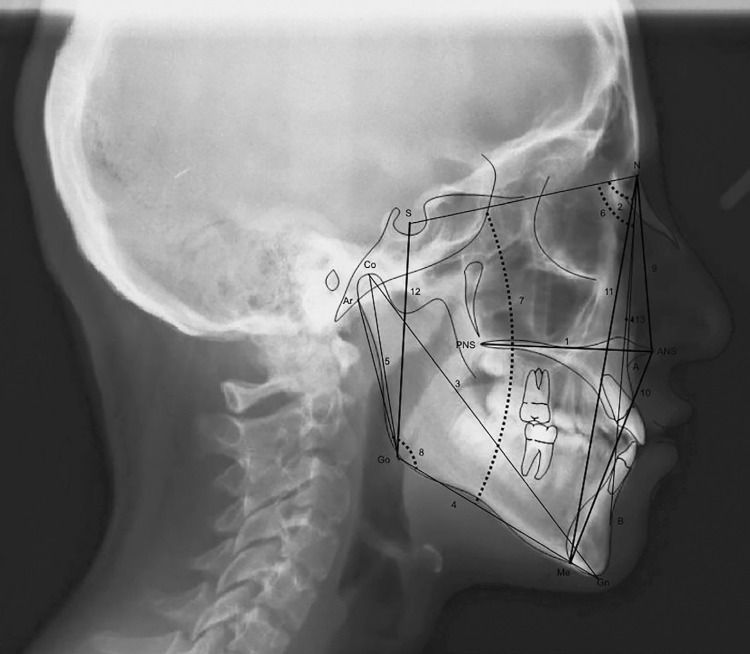
Maxillary, mandibular and maxillomandibular measurements. 1.ANSPNS(mm) 2.SNA(°) 3.Co-Gn(mm) 4.Go-Me(mm) 5.Co- Go(mm) 6.SNB(°) 7.GoGnSn(°) 8.Gonial angle(°) 9.N-ANS (mm) 10.ANS-Me(mm) 11.N-Me(mm) 12.S-Go(mm) 13.ANB(°)

**Figure 3:**
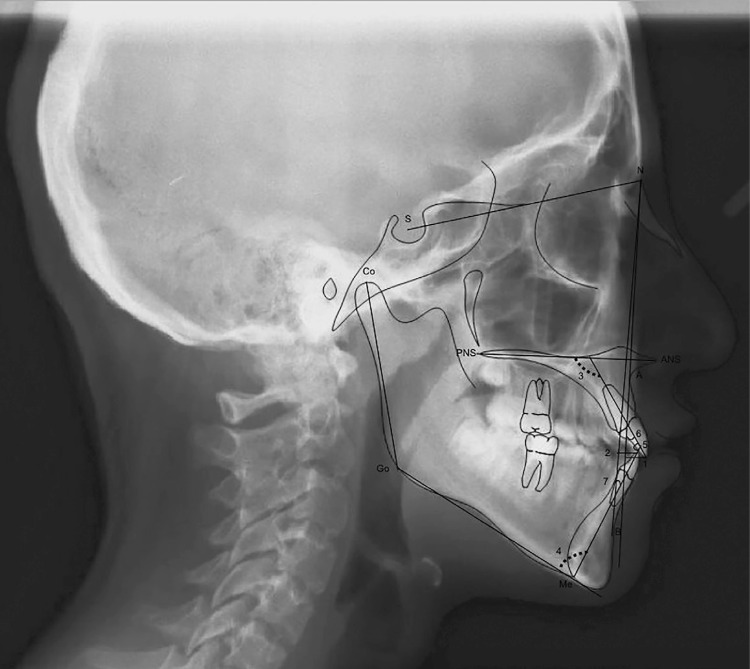
Dentoalveolar measurements. 1. U1-NA(mm) 2.L1-NB(mm) 3.U1-PP(°) 4.IMPA(°) 5.U1-L1(°) 6.U1-NA(°) 7.L1-NB(°)

**Figure 4:**
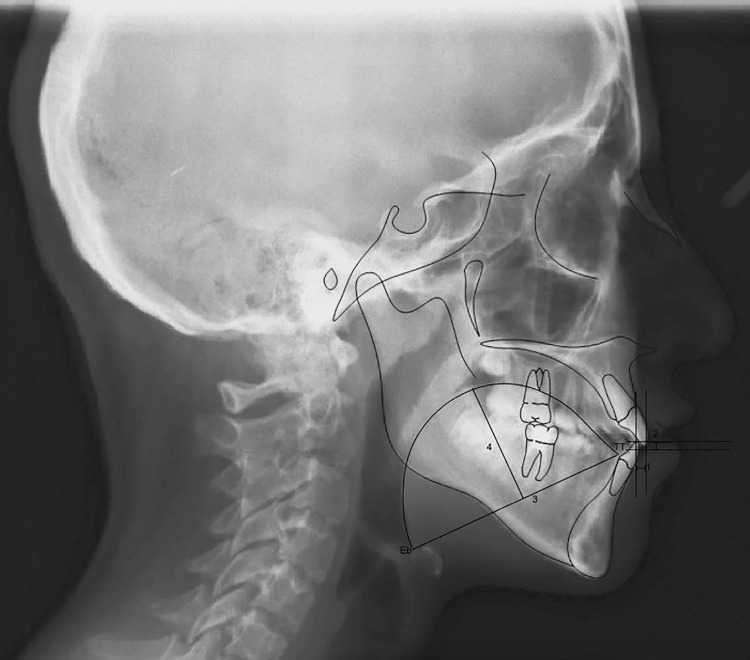
Tongue measurements, overjet and overbite. 1. Overjet(mm) 2.Overbite(mm) 3. Tongue length (mm) 4.Tongue height (mm)

**Figure 5:**
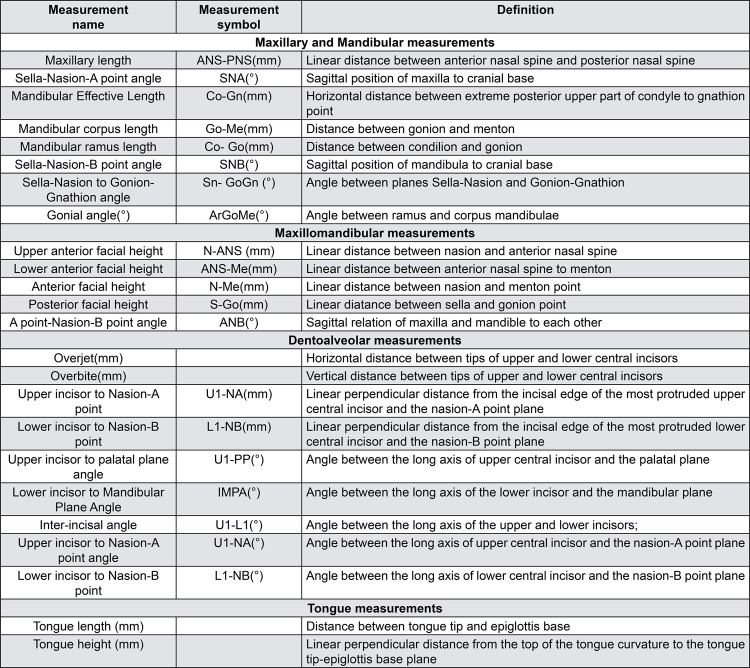
Cephalometric measurements used in this study

Routine radiographs taken before orthodontic treatment were used for evaluation. All cephalometric radiographs were traced by a single investigator (G.B.B.) using the Dolphin software (version 10.5, Patterson Dental Supply, St. Paul, MN, USA). Twenty-four measurements, commonly used and reported in the orthodontic literature, were taken on each cephalometric radiograph ( Figures 2 , 3 and 4 ). The measurements used in this study are shown in Figure 5 .

### Error assessment

To measure method error, 10 cephalograms (A point-Nasion-B point angle, Sella-Nasion to Gonion-Gnathion angle and upper incisor to palatal plane angle) and 10 sound recordings were randomly selected and reassessed by the same investigator (G.B.B.) after a 15-day interval. Consistency between repeated measurements was determined using the intraclass correlation coefficient, which ranged from 0.86 to 0.99. These values indicated that the measurements were reproducible and reliable.

### Statistical analysis

The Shapiro–Wilk test was used to determine whether the data exhibited normal distribution. The Mann–Whitney U test was used to compare characteristics between two independent groups when the data did not exhibit normal distribution; the Kruskal–Wallis test and all-pairwise multiple comparison test were used to compare characteristics among three or more independent groups when the data did not exhibit a normal distribution. Consistency of measurements taken at different intervals was assessed using the intraclass correlation coefficient. Relationships between numeric variables were tested using Spearman’s correlation coefficient. IBM SPSS Statistics 22 (IBM Corporation, NY, USA) was used for statistical analysis, and significance was considered when p<0.05.

## Results

The median values from the groups are shown in Table 1 . Center of gravity of the /s/ sound had the lowest value in Group 2 and statistically different from the Group 1. For the /s/ sound in Group 3, moderate positive correlations were found between center of gravity and Sella-Nasion to Gonion-Gnathion angle (Sn-GoGn) (p<0.05, r=0.444), Lower incisor to Nasion-B point (L1-NB mm) (p<0.023, r=0.505), and Lower incisor to Nasion-B point angle (L1-NB°)(p<0.034, r=0.476) ( Table 2 ).

**Table 1:** Median values and intergroup comparisons of all parameters

	Group 1	Group 2	Group 3	1-2-3	1-2	2-3	3-1
	(n=20)	(n=20)	(n=20)				
	Median	Median	Median				
	[%25-%75]	[%25-%75]	[%25-%75]				
/s/							
Cog [Hz]	9483	8765	8956	0,027*	0.009†	NS	NS
	[8270-10546]	[7995-9616]	[7622-10044]				

Median [%25-%75]; p<0.05 statistically significant; NS: Not Significant

* Kruskal Wallis, † All-Pairwise Multiple Comparison Test

**Table 2:** Spearman correlation coefficients between cog values for the /s/ sound and cephalometric measurements p<0.05 r: Spearman correlation coefficient; * Moderate correlation (0,4<r<0,6)

	/s/					
	Group 1		Group 2		Group 3	
	(n=20)		(n=20)		(n=20)	
	cog		cog		cog	
	r	P	r	P	r	P
ANS-PNS(mm)	-0. 272	0.246	0.255	0.278	-0.184	0.437
SNA(°)	-0.101	0.673	0.332	0.153	0.167	0.482
Co-Gn(mm)	0.106	0.656	-0.224	0.342	-0.006	0.980
Go-Me(mm)	-0.284	0.225	0.131	0.582	-0.292	0.212
Co- Go(mm)	0.216	0.360	-0.223	0.345	-0.034	0.887
SNB(°)	-0.032	0.895	0.283	0.227	0.046	0.847
GoGn-Sn(°)	0.214	0.366	-0.254	0.280	**0.444**	0.05*
Gonial angle(°)	0.136	0.567	-0.032	0.895	0.180	0.448
N-ANS (mm)	0.023	0.925	-0.102	0.668	-0.017	0.945
ANS-Me(mm)	0.113	0.636	-0.317	0.173	0.229	0.332
N-Me(mm)	0.150	0.527	-0.411	0.072	0.126	0.596
S-Go(mm)	0.107	0.654	-0.325	0.162	0.057	0.811
ANB(°)	-0.429	0.059	0.113	0.636	0.404	0.077
Overjet(mm)	-0.077	0.746	0.013	0.957	0.102	0.668
Overbite(mm)	-0.479	0.324	-0.075	0.753	-0.409	0.073
U1-NA(mm)	0.294	0.208	-0.101	0.673	0.005	0.985
L1-NB(mm)	-0.055	0.818	-0.079	0.740	**0.505**	0.023*
U1-PP(°)	0.411	0.072	0.092	0.701	-0.005	0.985
IMPA(°)	-0.392	0.087	0.251	0.286	0.194	0.412
U1-L1(°)	-0.026	0.915	0.050	0.835	-0.357	0.122
U1-NA(°)	0.305	0.191	0.017	0.945	0.033	0.890
L1-NB(°)	-0.419	0.066	0.199	0.401	**0.476**	0.034*
Tongue length (mm)	0.123	0.604	-0.123	0.605	0.011	0.962
Tongue height (mm)	-0.346	0.135	-0.056	0.816	0.169	0.477

## Discussion

Class II and Class III malocclusion may have negative effects on articulation. ^2 , 8 , 10 - 12^ Because 80% of specific speech movements occur at the front of the mouth, a causal relationship between speech disorders and malocclusion appears reasonable. ^13^ A sibilant sound, such as /s/, can be affected by the presence of Class II malocclusion. ^3 , 4 , 9 , 11^ Individuals with class III malocclusion and those with Class II malocclusion generally exhibit similar defects in consonant production. ^9^

Many studies on sound acoustics have benefited from spectral moment measurements in the analysis of fricative sounds. ^14 - 18^ The average energy distribution from any point (beginning, middle, or end) of a fricative sound shows its center of gravity value. ^19^ In our study, spectral moment analysis was used to determine the fricative spectrum. During production of the /s/ sound, the tip of the tongue is located on the alveolar ridge. ^20^ The fricative spectrum depends on the size of the oral cavity; if a difference occurs in the narrowing area, a change occurs in its average distribution. ^21^ Jesus, et al. ^22^ (2014) reported that the location of /s/ articulation was more posterior in patients with class II malocclusion than in those with class I malocclusion. If /s/ were produced with more posterior articulation, the size of the oral cavity would increase and the center of gravity would decrease. ^18^ In our study, center of gravity values were lower in Group 2 than in groups 1 and 3.

Benediktsson ^23^ (1958) found that /s/ sound production was affected by the relationship between the incisors, as well as the positions of the tongue and mandible. George ^24^ (1983) evaluated mandibular movements during production of the /s/ sound and found that the mandible had a wide range of motion. To pronounce the /s/ sound ideally – in terms of acoustics – the anterior teeth should come to an edge-to-edge position, dental arches should be slightly separated with the protrusion of the mandible and the tip of the tongue should lie horizontally posterior to the lower arch. ^5^ Producing such a mandibular position could be difficult for patients with class II and III malocclusions.

In our study, the spectral center of gravity of the /s/ sound in different malocclusion types was investigated together with the correlation of cephalometric measurements. Our findings show moderate correlations of the center of gravity with Sella-Nasion to Gonion-Gnathion angle and Lower incisor to Nasion-B point values in patients with Class III malocclusion. These measurements are influenced by the lower incisor and mandibular position. As mentioned earlier, these structures are involved in the producing of the /s/ sound. Thus, the mandible and lower incisors may affect center of gravity values of the /s/ sound especially in class III malocclusion.”

These findings should not be considered to constitute evidence of a direct relationship between malocclusion and speech disorders. ^25^ Speech disorders may be present in individuals with normal occlusion and in those with malocclusion. ^26 , 27^ In our study, center of gravity values did not differ among groups and were not correlated with A point-Nasion-B point angle (ANB), overjet, or overbite measurements; we had expected that these parameters would be affected more robustly. These surprising results suggest that adaptive functions remain effective in the presence of malocclusion, as demonstrated in previous studies. ^10 , 23 , 28^ This study is the first to investigate correlations between center of gravity measurements and cephalometric measurements. In the context of dentistry, further acoustic studies are needed to better characterize relationships between malocclusion types and the acoustic properties of speech sounds. Furthermore, in the light of the findings of this article, studies evaluating the effect of dental treatment on sound production are recommended.

The main limitation of this study is the lack of a standard audiometric examination. It has been generally accepted that high frequencies due to exposure to amplified music causes noise-induced hearing loss, which is more common among adolescents and young adults. ^29 - 31^ In the age group studied, there is a possibility of hearing loss.

## Conclusions

In conclusion, this study showed that the /s/ sound is affected by malocclusion due to change in articulation points. Moderate positive correlations were found between center of gravity and Sella-Nasion to Gonion-Gnathion angle, lower incisor to Nasion-B point and lower incisor to Nasion-B point angle in patients with class III malocclusion. No correlation was found in other cephalometric measurements. Our findings show that it is important to refer to orthodontic treatment to patients especially with class III in the early period. One can suppose that the orthodontic treatment may conduce to acoustically-ideal sound production by changing the place of articulation of the sounds.
